# Feasibility, Acceptability, and Influence of mHealth-Supported N-of-1 Trials for Enhanced Cognitive and Emotional Well-Being in US Volunteers

**DOI:** 10.3389/fpubh.2020.00260

**Published:** 2020-06-25

**Authors:** Richard L. Kravitz, Adrian Aguilera, Elaine J. Chen, Yong K. Choi, Eric Hekler, Chris Karr, Katherine K. Kim, Sayali Phatak, Sayantani Sarkar, Stephen M. Schueller, Ida Sim, Jiabei Yang, Christopher H. Schmid

**Affiliations:** ^1^Division of General Medicine, UC Davis Health, Sacramento, CA, United States; ^2^School of Social Welfare, University of California, Berkeley, Berkeley, CA, United States; ^3^WNYC Public Radio, New York, NY, United States; ^4^Betty Irene Moore School of Nursing, University of California, Davis, Sacramento, CA, United States; ^5^Center for Wireless & Population Health Systems, Qualcomm Institute, Department of Family Medicine & Public Health, Design Lab, University of California, San Diego, San Diego, CA, United States; ^6^Audacious Software, Inc., Chicago, IL, United States; ^7^College of Health Solutions, Arizona State University, Tempe, AZ, United States; ^8^Department of Psychological Science, University of California, Irvine, Irvine, CA, United States; ^9^Department of Medicine, University of California, San Francisco, San Francisco, CA, United States; ^10^Department of Biostatistics and Center for Evidence Synthesis in Health, School of Public Health, Brown University, Providence, RI, United States

**Keywords:** N-of-1 trial, single patient trial, mobile health, digital health, behavioral health, psychological well-being

## Abstract

Although group-level evidence supports the use of behavioral interventions to enhance cognitive and emotional well-being, different interventions may be more acceptable or effective for different people. N-of-1 trials are single-patient crossover trials designed to estimate treatment effectiveness in a single patient. We designed a mobile health (mHealth) supported N-of-1 trial platform permitting US adult volunteers to conduct their own 30-day self-experiments testing a behavioral intervention of their choice (deep breathing/meditation, gratitude journaling, physical activity, or helpful acts) on daily measurements of stress, focus, and happiness. We assessed uptake of the study, perceived usability of the N-of-1 trial system, and influence of results (both reported and perceived) on enthusiasm for the chosen intervention (defined as perceived helpfulness of the chosen intervention and intent to continue performing the intervention in the future). Following a social media and public radio campaign, 447 adults enrolled in the study and 259 completed the post-study survey. Most were highly educated. Perceived system usability was high (mean scale score 4.35/5.0, SD 0.57). Enthusiasm for the chosen intervention was greater among those with higher pre-study expectations that the activity would be beneficial for them (*p* < 0.001), those who *obtained* more positive N-of-1 results (as directly reported to participants) (*p* < 0.001), and those who *interpreted* their N-of-1 study results more positively (*p* < 0.001). However, reported results did not significantly influence enthusiasm after controlling for participants' interpretations. The interaction between pre-study expectation of benefit and N-of-1 results interpretation was significant (*p* < 0.001), such that those with the lowest starting pre-study expectations reported greater intervention enthusiasm when provided with results they interpreted as positive. We conclude that N-of-1 behavioral trials can be appealing to a broad albeit highly educated and mostly female audience, that usability was acceptable, and that N-of-1 behavioral trials may have the greatest utility among those most skeptical of the intervention to begin with.

## Introduction

Accumulating evidence supports the adoption of various habits and behaviors to improve cognitive and emotional well-being. For example, Americans are urged to be more physically active, reduce stress, and connect socially (1–5). One problem with the plethora of recommendations is that individuals may be confused about which behaviors to adopt first. They can turn to trusted experts, but most of the evidence upon which those experts rely is based on studies that generate average effects. Evidence derived from groups may not necessarily apply to the individual because of heterogeneity in person-level and contextual factors (e.g., age, gender, personal preferences, community resources, and fit with a person's life or workflow) (6, 7). Furthermore, the impact of any behavior is likely to yield modest benefits, potentially accumulating over time. More precise information on the likelihood of benefit *at the individual level* could help motivate long term behavior change.

Certainly, many people can and do assess the personal value of behavioral interventions informally through trial and error. Some, however, may be interested in a more rigorous approach. N-of-1 trials are multiple crossover trials conducted in a single individual (8). While sharing some characteristics with informal “trials of therapy,” they lend rigor to the assessment and, along with parallel group randomized controlled trials, are ranked at the top of the so-called evidence hierarchy by experts (9, 10). They have been used extensively in clinical psychology and medicine (11–17). For fast-acting, short-lived behavioral interventions expected to influence near-term outcomes, N-of-1 trials are arguably the most direct method for inferring the effect of treatment on an individual. These trials may appeal to persons who wish to gain greater certainty that the behavioral intervention under consideration actually does (or does not) have benefit for them.

Despite their theoretical appeal, N-of-1 trials have gained limited traction among clinicians and the general public (18). Part of the reason may be that when implemented according to the highest scientific standards (which may include blinding, use of complex outcome measures, strict attention to adherence, etc.), many potential participants will decide that the likely benefits (in terms of insights and motivation) are simply not worth the trouble. However, we and others have demonstrated that the reach and feasibility of N-of-1 trials may be extended through use of mobile health (mHealth) technologies; in our own recent study of patients with chronic pain, 88% of patients starting an n-of-1 trial reported that the mobile app was “extremely or very helpful.”(19) Another barrier may be the absence of scalable tools that allow non-scientists to conduct systematic evaluations of behavioral interventions on themselves (20).

We conducted this study to determine whether an mHealth supported N-of-1 trial assessing simple behavioral interventions for improving short-term cognitive and emotional well-being was feasible and perceived as beneficial. Specifically, we asked:

- Will members of the general adult population participate in an mHealth-facilitated behavioral N-of-1 trial?
- How do participants rate the usability of the mHealth N-of-1 trial system?
- To what extent are participant's attitudes toward the intervention and intentions to persist with it influenced by trial participation? Specifically,
  – Upon trial completion, how is enthusiasm for the chosen intervention related to expectations of benefit from the intervention, to the N-of-1 results themselves (as reported to the patient in terms of the difference in outcomes on days assigned to the intervention vs. days on their usual routine), and to the participant's interpretation of their N-of-1 results?

In addressing these questions, we sought to learn more about the utility of N-of-1 trials, the ways in which such trials affect participants' subsequent attitudes and behavioral intentions, and their prospects for broader adoption by the medical and behavioral community.

## Methods

### Design overview

A national convenience sample of adult volunteers was recruited to engage in a 30-day single person (N-of-1) trial comparing the effects of one of four behavioral interventions on self-reported stress, focus, and happiness. Participants selected an intervention and were assigned for 30 days to randomly sequenced five-day periods performing the chosen activity and their “usual routine.” Outcome measures were collected via secure text messaging. This report focuses on the 259 subjects who completed a post-study survey. Ethics approval was granted through the UC Davis Institutional Review Board (**IRB ID 1255435-4)**.

### Eligibility and Recruitment

We promoted the study through social media and The Brian Lehrer Show (WNYC Public Radio). Potentially interested subjects were directed to the study website (studyofme.org), where they were given the opportunity to watch videos introducing the study and asked to select an activity of interest. Available activities included: (1) deep breathing meditation; (2) gratitude journaling; (3) physical activity; and 4) performing acts of kindness for strangers. Drawing on cognitive-behavioral techniques and positive psychology, activities were selected to be simple and easy to apply repeatedly (21–24). Volunteers were eligible if they were US adults > = 18 years, owned a smartphone or had regular access to the internet, and were interested in committing to a 30-day N-of-1 trial. In addition, subjects were encouraged to try an activity that they were not already doing, and if they were considering vigorous physical activity, they were advised to “first consult your doctor, especially if you have a chronic health problem, recurring injury, or are pregnant or nursing.”

### Baseline Survey

After confirmation of eligibility and provision of online informed consent, participants completed a baseline questionnaire asking for contact information (mobile phone number and valid email, both of which were deleted from the dataset prior to analysis); time zone (so that study reminders would go out at the right time of day); N-of-1 trial start date within the next 7 days; demographic information (ethnicity, race, gender, education level, and household size); and several questions concerning experience with self-tracking and interest in the chosen activity.

### N-of-1 Trial Design and Conduct

All participants had the opportunity to read text and view a video providing detailed instructions on their chosen activity. Computer-generated N-of-1 trial sequences (e.g., UAUAAU, where U indicates 5 days performing usual routine and A indicates 5 days performing the chosen activity) were issued for each subject beginning on their chosen start date and continuing for 30 consecutive days. We used 5-day treatment periods as a compromise between the dictates of behavioral science (which would favor longer periods, to allow for adequate ramp-up and wash-out) (8) and statistical power (which would favor a greater number of switches between treatments). Participants received a text message through the HealthySMS system (25–27) each evening asking for ratings of stress, focus, and happiness for the day just finished and announcing tomorrow's activity.

**Figure 1:**
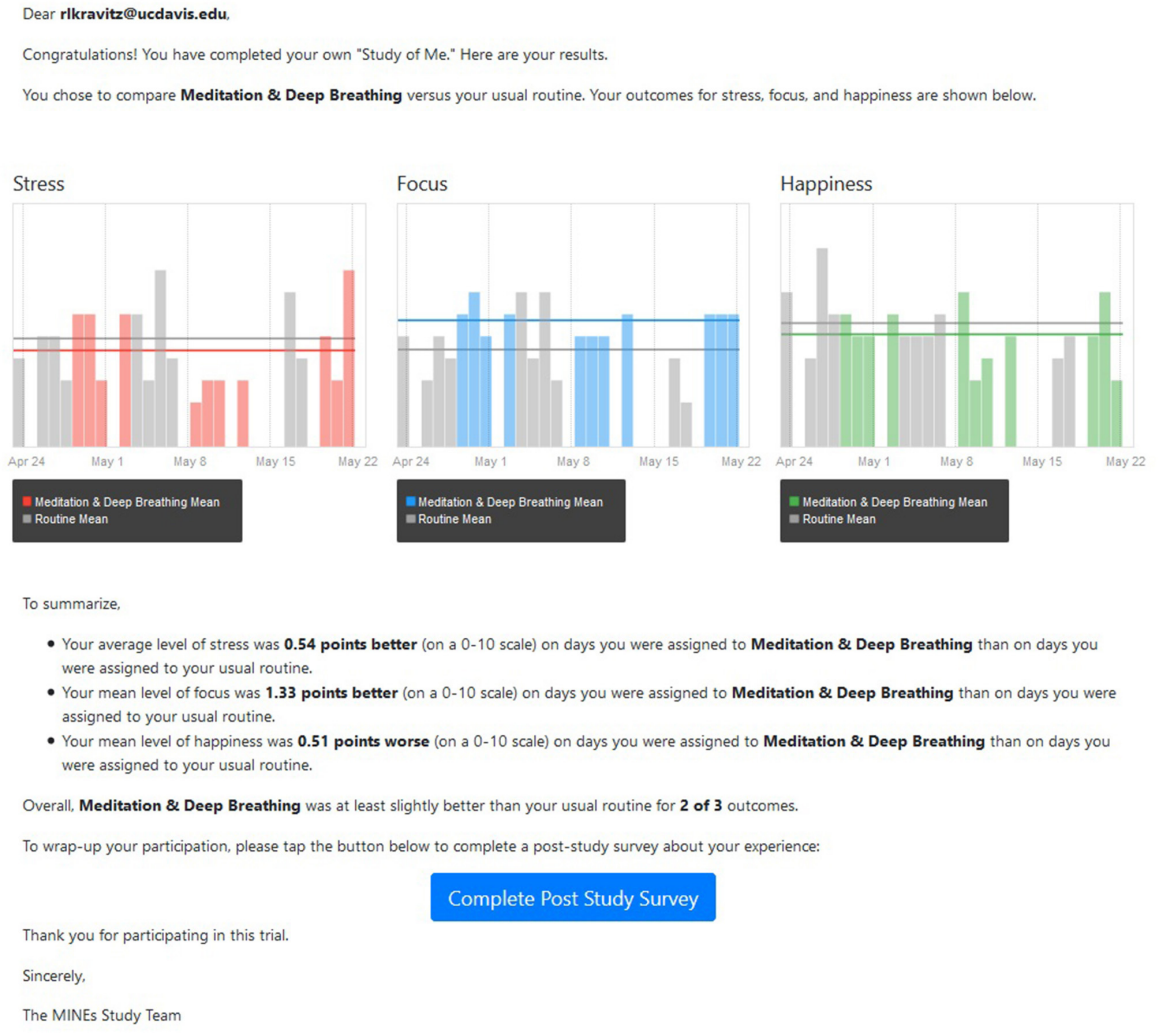
Sample participant results report. Participants in the study were provided with a both a graphic and a written summary depicting their gains (or losses) on days assigned to the intervention compared with days assigned to usual routine.

Within 1 week of N-of-1 trial completion, HealthySMS sent participants a text message with a link to their personalized results (example provided in Figure 1).

### Measures

Daily stress, focus, and happiness were each assessed with a single-item question sent each evening by text messaging: (1) On average, how stressed were you today? Please select a value from 0 (not at all stressed) to 10 (extremely stressed); (2) On average, how well were you able to focus? Please select a value from 0 (not able to focus at all) to 10 (extremely focused throughout the day); (3) On average, how happy were you today? Please select a value from 0 (not happy at all) to 10 (extremely happy throughout the day). Single-item measures of these constructs are typically used in studies that require daily responses by participants to reduce participant burden (28), and have demonstrated good reliability and validity when compared to longer measures (29–31).

At the end of the 30-day period, participants were sent a post-study questionnaire requesting completion of the System Usability Scale (32) (Cronbach's alpha in the sample, 0.83), and four questions assessing: (1) *pre-study expectations of benefit* of the chosen activity (“Before you started your personalized experiment, how confident were you that ACTIVITY would be beneficial for you?” 1 = not-at-all confident…5 = extremely confident); (2) *post-study* interpretation of results (“What is your best guess about what the RESULTS of your personalized experiment mean? 4 = highly beneficial, 3 = somewhat beneficial, 2 = minimally beneficial, 1 = not beneficial); (3) *post-study* perceptions of activity helpfulness (“Now that you have completed your personalized experiment, how helpful do you think ACTIVITY was for you? (1 = not at all helpful…5 = extremely helpful); and (4) *post-study* behavioral intentions (“Based on your personalized experiment, how likely are you to continue doing ACTIVITY on a regular basis over the next six months? 1 = not-at-all likely…5 = extremely likely).

We created an *Activity Enthusiasm Score* as the mean of perceived helpfulness of the activity (1–5 scale) and likelihood of continuing activity on a regular basis (1–5 scale), both measured after N-of-1 completion on a 1–5 scale. Cronbach's alpha for this 2-item index was 0.77, indicating acceptable to good internal consistency (33).

We summarized the *actual* results of each subject's N-of-1 trial in two ways. First, we directly evaluated differences in means for focus, stress, and happiness (each reported on a 0–10 scale) by taking the difference between the mean value during activity days and the mean value during the participant's usual routine, reversing the sign for stress, then summing across the three outcomes. The theoretical range of this scale was −30 to +30 and the actual range was −7.4 to 9.3. Second, we counted the number of outcomes (stress, focus, happiness) in which the mean value of the participant's responses while performing the chosen activity was better (more positive or less negative) than the mean value of the participant's response while performing their usual routine. Possible values of this count variable ranged from 0 (no outcome better, even by a small amount, during activity days) to 3 (all outcomes better during activity days). The difference variable accounts for the magnitude of benefit but does not consider precision (i.e., the metric does not take into account the within-individual variance in reported outcomes nor the number of measures reported by each participant. The count variable focuses on the number of outcome dimensions that were “improved,” while ignoring the magnitude of the improvements. We chose to evaluate these metrics rather than more sophisticated alternatives because they more closely comport with the data actually supplied to participants as shown, for example, in Figure 1). Because the results using the two metrics were not materially different, we report only the difference measure.

### Statistical Analysis

Values were expressed as means with standard deviations for continuous variables and counts with proportions for categorical variables. For characteristics of the analytic sample, analysis of variance (ANOVA) was performed to test whether there were differences in the means of continuous variables across different chosen activity groups. Chi-square test was performed to test for whether there was association between categorical variables and chosen activity groups if the minimum expected cell count was greater than 1; otherwise, Fisher's exact test was performed. The same procedure was also applied when comparing those who completed the post-study survey with those who did not.

Multiple linear regression was used to assess the relationship between Activity Enthusiasm Score and expectations of benefit from the intervention, interpretation of n-of-1 results, and actual reported results as represented by the summated score along with their pairwise interactions. Goodness-of-fit was expressed by the coefficient of determination, *R*^2^. A significant relationship was determined by a *p* < 0.05. Stata software version 15 was used for regression modeling. R software version 3.6.1 was used to produce graphs.

## Results

**Figure 2:**
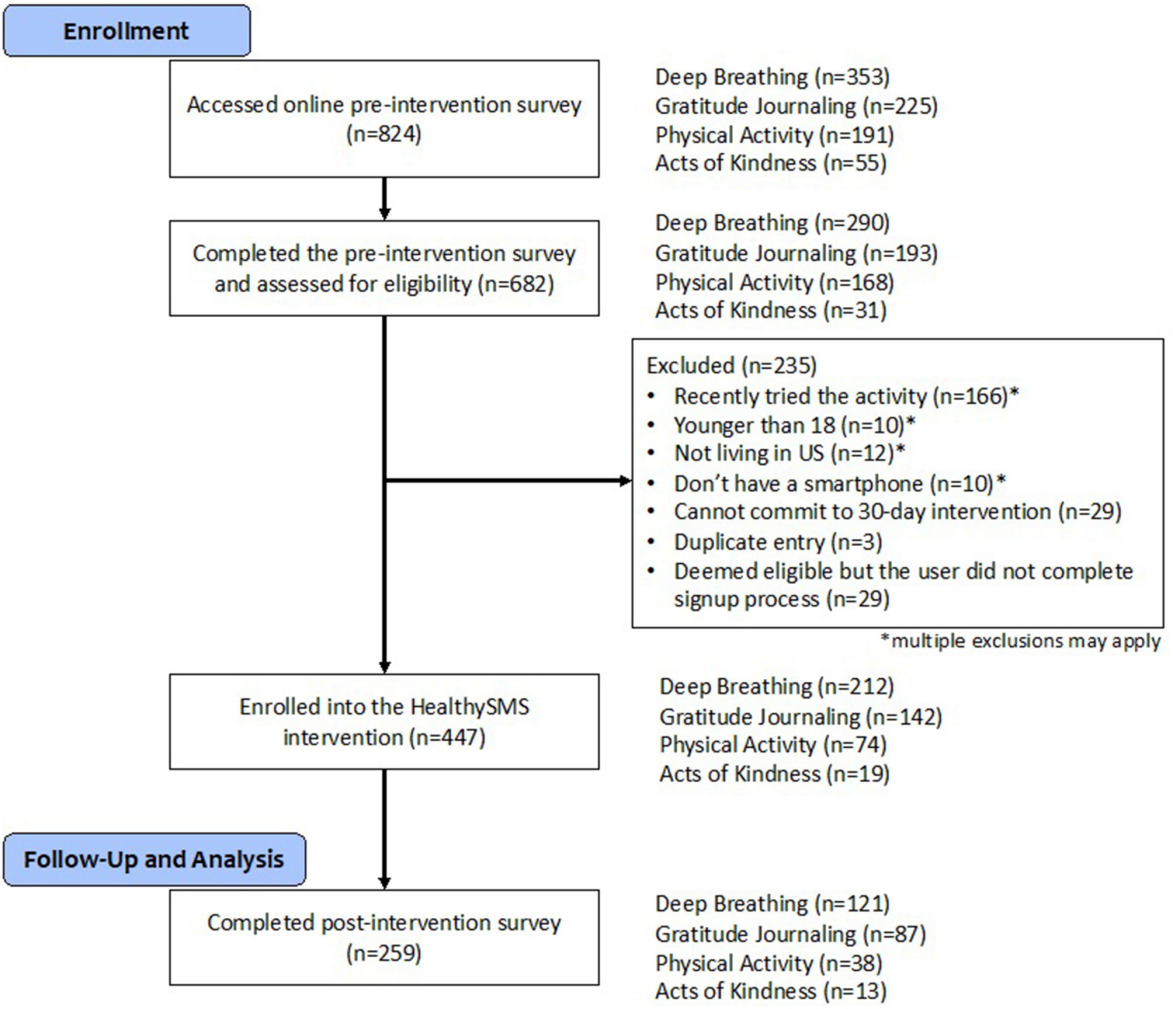
CONSORT diagram illustrating participant flow through the study.

Of 824 volunteers who *accessed* the online pre-study questionnaire (353 who selected deep breathing, 225 gratitude journaling, 191 physical activity, and 55 acts of kindness), 682 subjects completed the pre-study survey and were assessed for eligibility, 447 signed onto the HealthySMS platform, and 259 completed the post-study survey Figure 2. The mean proportion of daily assessments actually returned was 0.70 (SD 0.22).

Among the 259, the mean age was 51 and most were from the Eastern time zone, female, white, and very highly educated; a minority lived alone or had previously tried self-experimentation (Table 1) There were no significant associations between respondents' personal characteristics and the behavior intervention activity they chose to evaluate (Table 1). There were no meaningful or statistically significant demographic differences between the 259 participants included in the analytic sample and the remaining 188 participants who enrolled in the study but did not complete any part of the post-study questionnaire, except that completers were about 4 years older (Supplemental Material Table 1).

**Table 1:** Characteristics of analytic sample, overall, and by chosen activity.

**Characteristic**	**Overall (*n* = 259)**	**Deep breathing (*n* = 121)**	**Gratitude journaling (*n* = 87)**	**Physical activity (*n* = 38)**	**Acts of kindness (*n* = 13)**	***P*-value**
Age, yrs. (SD)	50.5 (13.9)	51.8 (14.0)	48.4 (13.4)	50.7 (14.4)	51.5 (14.7)	0.36
Time zone, *n* (%)						0.44
Eastern	148 (57.1)	75 (62.0)	46 (52.9)	21 (55.3)	6 (46.2)	
Central	44 (17.0)	17 (14.0)	17 (19.5)	6 (15.8)	4 (30.8)	
Mountain	20 (7.7)	8 (6.6)	9 (10.3)	1 (2.6)	2 (15.4)	
Pacific	47 (18.1)	21 (17.4)	15 (17.2)	10 (26.3)	1 (7.7)	
Female, *n* (%)	219 (84.6)	107 (88.4)	69 (79.3)	33 (86.8)	10 (76.9)	0.27
Nonwhite (including mixed), *n* (%)	41 (15.8)	20 (16.5)	11 (12.6)	8 (21.1)	2 (15.4)	0.69
Education						0.21
<Bachelor's degree, refused, or other	52 (20.1)	25 (20.7)	11 (12.6)	11 (28.9)	5 (38.5)	
Bachelor's degree	66 (25.5)	31 (25.6)	26 (29.9)	7 (18.4)	2 (15.4)	
Advanced degree	141 (54.4)	65 (53.7)	50 (57.5)	20 (52.6)	6 (46.2)	
Lives alone, *n* (%)	44 (17.0)	14 (11.6)	16 (18.4)	10 (26.3)	4 (30.8)	0.08
Previously tried self-experimentation, *n* (%)	47 (18.1)	25 (20.7)	17 (19.5)	4 (10.5)	1 (7.7)	0.38

Most respondents strongly or somewhat agreed with positive statements about system usability and strongly or somewhat disagreed with negative statements (Table 2). The mean scale score was 4.35 (SD 0.57) (Table 2), corresponding to a percentage-based score of 4.35/5 × 100 = 87.1, well above the average of 68 previously reported and comparable to microwave ovens, which received a rating of 87 in a consumer survey of 1,058 participants (34, 35). System Usability Scale scores were lower, on average, among participants choosing Acts of Kindness compared to those choosing Deep Breathing or Physical Activity (*p* = 0.008, with pairwise comparisons between Acts of Kindness and both Deep Breathing and Physical Activity significant using the Bonferroni approach (*p* < 0.01 in each case, data not shown in tabular form). However, there were no significant differences in System Usability Scale scores by age, gender, race, or education (see Supplemental Material Table 2 for details).

**Table 2:** Respondents' experiences with N-of-1 trial system usability (*n* = 252)^*^.

**Item**	**Strongly or somewhat agree, *n* (%)**	Item mean^*^ (SD)
I think I would like to use this system frequently	144 (55.6)	3.5 (1.1)
I found the StudyofMe system unnecessarily complex	10 (3.9)	1.5 (0.9)
I thought the StudyofMe system was easy to use	233 (90.0)	4.6 (0.8)
I think that I would need the support of a technical person to be able to use the system	6 (2.3)	1.2 (0.6)
I found the various functions in the StudyofMe system were well-integrated	156 (60.2)	3.8 (1.1)
I thought there was too much inconsistency in the StudyofMe system	26 (10.0)	1.8 (1.1)
I would imagine that most people would learn to use the StudyofMe system very quickly	237 (91.5)	4.6 (0.7)
I found the StudyofMe system very cumbersome to use	24 (9.3)	1.6 (1.1)
I felt very confident using the StudyofMe system	209 (80.7)	4.4 (1.0)
I needed to learn a lot of things before I could get going with the StudyofMe system	5 (1.9)	1.3 (0.7)
System usability scale	–	4.35 (0.57)^⊥^

* *N = 252 rather than 259 because 7 subjects did not complete a majority of scale items*.

⊥ *In calculating the mean scale score, items with negative valence were reversed*.

On the post-study questionnaire, 32% of respondents recalled that prior to starting the N-of-1 trial they were very or extremely confident that the chosen activity would be beneficial. At the same time, 27 (10%) interpreted their reported N-of-1 results as indicating that the activity was *not* beneficial for them, 77 (30%) that the activity was *minimally* beneficial, 119 (46%) that the activity was *somewhat* beneficial, and 25 (10%) that the activity was *highly* beneficial. Participants with positive expectations for intervention benefit (i.e., those who reported being “very” or “extremely” confident that the chosen intervention would be beneficial) were more likely than their more skeptical peers to interpret their results as showing that the activity was “somewhat” or “highly” beneficial (68 vs. 53%, *p* = 0.036, data not shown in tabular form).

Table 3 examines the effects of pre-study expectations for benefit, the participant's interpretation of their own N-of-1 results, and actual reported results (as represented by the difference metric; see Methods) on Activity Enthusiasm Score. Model 1 shows that both expectations for intervention benefit (*p* < 0.001) and interpretation of own results (*p* < 0.001) were significantly associated with enthusiasm for the chosen activity, accounting for 33% of the variance. Substituting *actual* results for *interpretation* of results resulted in a regression (Model 2) that explained only 14% of the variance in enthusiasm. Finally, actual results were not significantly associated with enthusiasm after adjusting for pre-study confidence and results interpretation (Model 3).

**Table 3:** Influence of pre-study confidence, interpretation of own results, and actual (reported) results on participant's “enthusiasm” for the behavioral intervention^£^.

	**Model 1 (pre-study confidence and interpretation of own results, without interaction) (*****n*** **=** **248)**		**Model 2 (pre-study confidence and actual results) (*****n*** **=** **245)**		**Model 3 (pre-study confidence, interpretation of own results, and actual results) (*****n*** **=** **245)**	
Predictor variable	Coefficient	95% CI	Coefficient	95% CI	Coefficient	95% CI
Pre-study confidence (1–5 scale)	0.20	(0.10, 0.30)^*^	0.27	(0.16, 0.38)^*^	0.20	(0.10, 0.30)^*^
Interpretation of own results (1–4 scale)	0.54	(0.43, 0.65)^*^	–		0.48	(0.36, 0.60)^*^
Actual results^†^	–	–	0.10	(0.05, 0.14)^*^	0.03	(-0.01, 0.07)
R-squared	0.33		0.14		0.31	

£ All models in this table use multiple linear regression to estimate “enthusiasm” as a function of various predictors. As described in Methods, “enthusiasm” is an index ranging from 1 (low) to 5 (high) combining perceived “helpfulness” of the intervention and likelihood of persisting with the intervention over the next 6 months. Model 1 examines the influence on enthusiasm for the intervention (1–5 scale) of pre-study confidence (1 = not-at-all confident…5 = extremely confident) and the participant's interpretation of their own n-of-1 results (1 = intervention not beneficial…4 = intervention extremely beneficial). In a variation of this model (not shown), the interaction of confidence and results interpretation was significant with a negative sign, indicating that interpretation of own results was a more potent predictor of enthusiasm among those with lower pre-study confidence. However, this model is not further considered for ease of interpretation. Model 2 uses multiple linear regression to estimate the effects on enthusiasm of confidence and actual n-of-1 study results as reported to the participant (using the “difference measure” as defined in Methods, actual range −7.4 to 9.3), and Model 3 evaluates confidence (1–5 scale), interpretation of results (1–4 scale), and actual results (difference measure). Interaction terms are not reported for Models 2 and 3 because preliminary analysis showed no significant contribution of any two-way interaction.

† *Average of the mean difference between intervention and control rating stress, focus, and happiness. Scores for stress were reversed so that a more positive difference between intervention and control consistently represents a better outcome*.

* *p < 0.001*.

**Figure 3:**
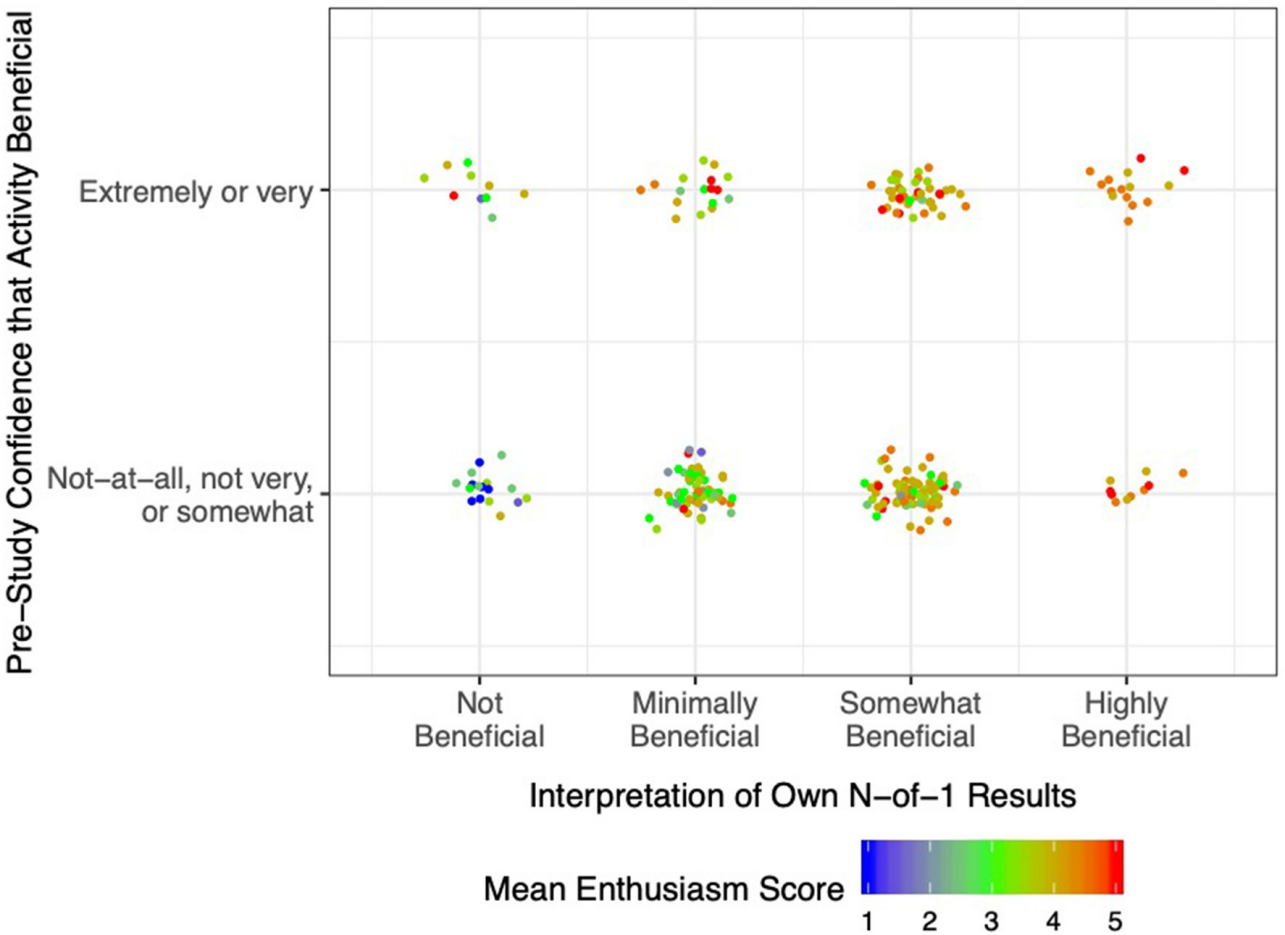
Post-Study Enthusiasm Scores (mean of perceived intervention helpfulness and likelihood of persisting with the activity in future) as a function of pre-study confidence in the activity (reported retrospectively) and interpretation of own n-of-1 trial results. Each dot represents a single participant, with warmer colors indicating greater enthusiasm.

The relationship of pre-study expectations for benefit, post-study interpretation of results, and Activity Enthusiasm Score is further illustrated in Figure 3. Essentially, if at the outset respondents were highly confident that their chosen activity was beneficial (top row), enthusiasm remained moderate to high regardless of actual study results (plenty of orange and red dots, and very few blue dots, even among participants who interpreted their own n-of-1 results as showing that the activity delivered little to no benefit). On the other hand, if initial confidence for benefit was low-to-moderate (bottom row), enthusiasm was more strongly related to the participant's interpretation of their own results (mostly blue dots in the “not beneficial” column, mostly red dots in the “highly beneficial” column). These results are replicated in tabular form in Supplemental Material Table 3.

## Discussion

As the most direct approach to estimating individual treatment effects, N-of-1 trials have been called the holy grail of clinical investigation (36). The method's appeal may also extend to selected lay audiences, such as the quantified-self movement (37). Broader uptake of N-of-1 trials could help people with and without chronic diseases to more quickly identify treatments or lifestyle interventions that are both appealing and effective. However, logistical barriers, technical concerns, and simple lack of awareness have impeded dissemination and uptake. The main contribution of the current study is to demonstrate that N-of-1 trials of behavioral interventions can attract substantial interest from a relatively broad cross-section of US adults. However, participants were highly educated and tilted strongly female. There are several possible explanations for limited participation among men and those without a college degree, including relative indifference to the topic of “wellness;” competing demands from other responsibilities; or a persistent “digital divide” curtailing access or limiting comfort with mobile devices (38). Nevertheless, our mHealth platform supporting these trials was rated highly usable by participants. Finally, participants' *a priori* expectations for benefit of their chosen behavioral intervention (as measured by confidence that the activity would be beneficial) as well as their *a posteriori* interpretation of their N-of-1 trial results were both significant independent predictors of enthusiasm for the intervention going forward.

We recruited participants using social media (principally Facebook) plus an on-air interview with WNYC Public Radio host Brian Lehrer. Over a brief recruitment period, 824 individuals demonstrated interest by visiting the study website, but unsurprisingly, there was significant attrition at every stage thereafter. Among the 259 subjects in the analytic cohort, the modal participant was a white, middle-aged, highly educated woman. However, less than one in five had prior experience with self-experimentation, indicating both that our sample was open to novel experiences and that N-of-1 trials may have appeal beyond the established self-tracking community.

Participants generally rated the HealthySMS platform as highly accessible and easy to use without technical support, despite modest misgivings about functional integration. These findings are especially remarkable in light of the native complexity of N-of-1 trials. For example, in our study, patients needed to become comfortable with a new behavioral intervention, switch off regularly between the intervention and their usual routine, and report daily ratings of stress, focus, and happiness.

Pre-study expectations for benefit from the chosen behavioral intervention was modestly associated with post-study enthusiasm for the intervention. At the same time, participants who interpreted their N-of-1 results as highly beneficial had much greater enthusiasm than those who interpreted their results as indicating that the intervention was not beneficial. However, the effect of participants' interpretations of their own results on enthusiasm for the intervention was greater among those with the least confidence in the intervention to begin with.

One interpretation of these findings is that N-of-1 trials had greater information value for participants who were more skeptical at the outset; in Bayesian terms, those with weak or negative priors relied more on the incoming data (39). Although considerable work in cognitive psychology indicates that humans are poor Bayesians (40), our results suggest that in the context of a self-experiment in which they are personally vested, participants may form conclusions based on a weighted average of pre-trial expectations and post-trial results. A plausible implication is that investigators should explicitly account for participants' prior beliefs in the context of N-of-1 experiments and, indeed, use them in constructing posterior probabilities that are returned to patients. Another possible explanation, drawing on expectation disconfirmation theory (41), is that participants who were pleasantly surprised by a positive result (despite expecting a negative outcome) were more likely to be enthused about the activity going forward.

Although participants' actual results (as conveyed by a graphical interface supported by text, as in Figure 1) were moderately correlated with their subjective interpretations, the former did not significantly predict intervention enthusiasm after adjusting for the latter, suggesting that actual results are mediated through participants' interpretations. Furthermore, participants' interpretations may not fully and accurately incorporate actual results—even among the highly educated. More work is needed on ways to accurately convey n-of-1 results to participants, especially in real-world, non-clinical settings where clinicians and investigators are unavailable to help.

The strengths of this study include attention to several novel questions and the use of innovative methods to attract participants and to support them in conducting their own single-patient trials. However, as with all studies, the findings must be evaluated in light of certain limitations. First, there was substantial attrition between expressing initial interest and completing a minimum number of study procedures. Second, the analytic sample was demographically narrow, limiting generalizability (42). This likely reflected some combination of our outreach methods (social media and public radio, which may appeal to a more socio-economically advantaged cohort); the “digital divide;” and the intrinsic appeal of “wellness” interventions and self-monitoring to certain demographic groups (e.g., women). Third, in measuring daily outcomes with single items, we likely sacrificed reliability in the interest of minimizing respondent burden. Fourth, measuring pre-study confidence in the benefits of the intervention after participants completed their N-of-1 trial could have introduced recall or “hindsight” bias. In retrospect, it would have been preferable to measure expectations prospectively, and future studies should do this. Hindsight bias would tend to narrow the gap between participants' expectations and *post-hoc* enthusiasm for the intervention. Had we measured expectations prospectively, we might have seen a more consistent gap in enthusiasm between those with high and low expectations. Finally, we made no attempt to measure either behavior change or psychological outcomes beyond 4–6 weeks after the start of each participant's N-of-1 trial.

In summary, this study demonstrates that N-of-1 trials can be disseminated to a broad, if demographically slanted, subset of the general US population in the interest of enhancing psychological well-being. Subjects appear to learn from their own N-of-1 experiences, although their learnings are tempered by prior beliefs. Our finding of increased influence of trial results among those with the lowest a priori expectations of benefit suggests that mHealth-supported, behavioral N-of-1 trials may have the greatest value for those with the lowest outcomes expectations; these individuals may be exactly those with more health problems and higher need. Further research is needed to clarify who can benefit from such trials, under what circumstances, and with respect to which medium and long-term outcomes.

## Data Availability Statement

The datasets generated and analyzed for this study can be accessed by writing to the corresponding author.

## Ethics Statement

The studies involving human participants were reviewed and approved by the UC Davis Institutional Review Board (IRB ID 1255435-4). The patients/participants provided their written informed consent to participate in this study.

## Author Contributions

RK conceived of the study, obtained funding, performed selected analyses, and wrote the first draft of the manuscript. AA, CK, KK, and SS supported the technical implementation of the study, participated in data collection, and edited the manuscript. EC, EH, SP, SMS, and IS conceived of the study, contributed to design and implementation, and edited the manuscript. YC, JY, and CS performed and/or supervised the statistical analysis and edited the manuscript. All authors contributed to the article and approved the submitted version.

## Conflict of Interest

CK is the founder of Audacious Software LLC and provided software development, design, and support services for research efforts. AA is (together with University of California, Berkeley) the owner of the HealthySMS license. Fees are paid to UC Berkeley for its use. EH is scientific advisor to Fitabase. KK is an advisor to Bluenote Therapeutics. SS is a consultant with Otsuka Pharmaceutical. IS is a member of the Medical Advisory Board for 98point6; has stock options in Myia Labs, Inc.; is a scientific advisor and consultant for Myovant; and is a Board member and consultant at Vivli. The remaining authors declare that the research was conducted in the absence of any commercial or financial relationships that could be construed as a potential conflict of interest.
