# Supplementary material for: Feasibility, Acceptability, and Influence of mHealth-Supported N-of-1 Trials for Enhanced Cognitive and Emotional Well-Being in US Volunteers
Source: Front Public Health. 2020 Jun 25;8:260. doi: 10.3389/fpubh.2020.00260 (PMC7336867; doi:10.3389/fpubh.2020.00260)
Supplement: Supplementary file 1 [file Table_1.docx]

Appendices

**Appendix Table 1**. Comparison of subjects completing post-study questionnaire with those enrolled in study but not completing post-study questionnaire.

| Characteristic | Post-study Questionnaire Completed  (n = 259) | Post-study Questionnaire Not Completed  (n = 188) | P-value |
| --- | --- | --- | --- |
| Age, yrs. (SD) | 50.5 (13.9) | 46.1 (13.4) | <0.01 |
| Time zone, n (%)  Eastern  Central  Mountain  Pacific  Alaska  Hawaii | 148 (57.1)  44 (17.0)  20 (7.7)  47 (18.1)  0 (0.0)  0 (0.0) | 102 (54.3)  30 (16.0)  16 (8.5)  38 (20.2)  1 (0.5)  1 (0.5) | 0.73 |
| Female, n (%) | 219 (84.6) | 155 (82.4) | 0.64 |
| Nonwhite, n (%) | 41 (15.8) | 38 (20.2) | 0.28 |
| Education  <BS/BA  BS/BA  Advanced degree | 52 (20.1)  66 (25.5)  141 (54.4) | 33 (17.6)  65 (34.6)  90 (47.9) | 0.11 |
| Lives alone, n (%) | 44 (17.0) | 33 (17.6) | 0.98 |
| Previously tried self-experimentation, n (%) | 47 (18.1) | 42 (22.3) | 0.33 |

**Appendix Table 2**. Respondents’ experiences with N-of-1 trial system usability by participants’ characteristics.

| Characteristic | Average System Usability Scale Scores (SD) | P-value |
| --- | --- | --- |
| Age, yrs. (Coefficient from linear regression presented) | 3.59$\times{10}^{-3}$ (2.62$\times{10}^{-3}$) | 0.17 |
| Female  Yes  No | 4.38 (0.57)  4.21 (0.54) | 0.08 |
| Nonwhite  Yes  No | 4.25 (0.63)  4.37 (0.56) | 0.23 |
| Education  <BS/BA  BS/BA  Advanced degree | 4.46 (0.51)  4.23 (0.72)  4.37 (0.51) | 0.09 |

**Appendix Table 3.** Post-study *enthusiasm* for the chosen activity as a function of pre-study confidence that the chosen activity would be beneficial and post-study interpretation of own N-of-1 trial results. (n=248)*

|  | **Mean Enthusiasm Score (SD)** | | | |  |
| --- | --- | --- | --- | --- | --- |
|  | **Interpretation of Own N-of-1 Results** | | | |  |
| **Pre-Study Confidence that Activity Beneficial** | Not Beneficial (n=27) | Minimally Beneficial (n=77) | Somewhat Beneficial (n=119) | Highly Beneficial (n=25) | Total (n=248) |
| Extremely or very (n=84) | 3.4 (0.97) | 3.8 (0.81) | 4.1 (0.56) | 4.4 (0.32) | 4.0 (0.70) |
| Not-at-all, not very, or somewhat (n=164) | 2.2 (1.08) | 3.4 (0.80) | 3.9 (0.61) | 4.5 (0.41) | 3.6 (0.91) |
| Total (n=248) | 2.7 (1.2) | 3.5 (0.82) | 4.0 (0.60) | 4.5 (0.35) | 3.7 (0.87) |

*n=248 because 11 subjects had missing values for one or more variables in this analysis. P-values were generated through analysis of variance. The main effect of “interpretation of own results”on intervention enthusiasm was significant, p<0.001. The main effect of “pre-study confidence” on intervention enthusiasm was also significant, p<0.001. There was a significant interaction between pre-study confidence and interpretation of own results, p=0.009.
